# The International Science and Evidence-based Education Assessment

**DOI:** 10.1038/s41539-021-00085-9

**Published:** 2021-03-01

**Authors:** Anantha Duraiappah, Nienke van Atteveldt, Stanley Asah, Gregoire Borst, Stephanie Bugden, J. Marieke Buil, Oren Ergas, Stephen Fraser, Julien Mercier, Juan Felipe Restrepo Mesa, Alejandra Mizala, Yoko Mochizuki, Kaori Okano, Christopher Piech, Kenneth Pugh, Rajiv Ramaswamy, Nandini Chatterjee Singh, Edward Vickers

**Affiliations:** 1 UNESCO Mahatma Gandhi Institute of Education for Peace and Sustainable Development (MGIEP), New, Delhi, India; 2 Department of Clinical, Neuro- and Developmental Psychology & Institute Learn!, Vrije Universiteit Amsterdam, Amsterdam, The Netherlands; 3 Human Dimensions of Natural Resource Management, School of Environmental & Forest Sciences, College of the Environment, University of Washington, Seattle, WA USA; 4 Laboratoire de Psychologie du Développement et de l’éducation de l’enfant (LaPsyDÉ - CNRS), Université de Paris, Paris, France; 5 Department of Psychology, The University of Pennsylvania, Philadelphia, PA USA; 6 Faculty of Education, Beit Berl College, Beit Berl, Israel; 7 Education Endowment Foundation, London, UK; 8 Département d’éducation et formation spécialisées, Université du Québec à Montréal, Montreal, QC Canada; 9 Colegio Montessori de Cartagena, Cartagena, Colombia; 10 Instituto de Estudios Avanzados en Educación, Universidad de Chile, Santiago, Chile; 11 Department of Languages and Linguistics, School of Humanities and Social Sciences, La Trobe University, Melbourne, VIC Australia; 12 Computer Science Department, Stanford University, Stanford, CA USA; 13 Haskins Laboratories, New Haven, CT USA; 14 Kyushu University, Fukuoka, Japan

**Keywords:** Policy, Education

## Abstract

Education is indispensable for the flourishing of people from all backgrounds and stages of life. However, given the accelerating demographic, environmental, economical, socio-political, and technological changes—and their associated risks and opportunities—there is increasing consensus that our current educational systems are falling short and that we need to repurpose education and rethink the organization of learning to meet the challenges of the 21st century. The United Nations Educational Scientific and Cultural Organization (UNESCO) “Futures of Education” initiative was formally launched at the United Nations General Assembly in 2019 to provide such a vision of education for the future. The International Scientific and Evidence-based Education (ISEE) Assessment synthesizes knowledge streams generated by different communities and stakeholders at all levels and scales and will thereby essentially contribute to re-envisioning this future of education. The overall aim of the ISEE Assessment is to pool the expertise from a broad range of knowledge holders and stakeholders to undertake a scientifically robust and evidence-based assessment in an open and inclusive manner of our current educational systems and its necessary reforms. In this commentary, we discuss the aims and goals of the ISEE Assessment. We describe how the ISEE Assessment will address key questions on the purpose of education and what, how, where and when we learn, and evaluate the alignment of today’s education and theory of learning with the current and forthcoming needs and challenges and to inform policymaking for future education.

Not many would argue with the claim that education matters, for people of all stages of life. However, there is less agreement on the purpose of education. Should it be to improve the human condition, or should it be directed toward meeting the demands of the workplace to promote economic growth? Is prosperity, as presently measured by gross domestic product (GDP), positively related to the state of education systems^1,2^? Moreover, the flourishing of today’s societies is challenged in different ways than was the case 300 years ago, when systems of mass schooling developed in tandem with the emergence of modern nation-states^3^. Climate change, uncertain job markets, growing social inequality, and pandemics such as the ongoing Covid-19, are the challenges we currently face. Our future more than ever depends on how we, as a global society, build our education systems to ensure continued human advancement and flourishing.

We start by asking two fundamental questions: are our education systems still serving the right purpose? And are they equipped to address the pressing challenges we face? To answer these questions and provide guidance on ways forward, an assessment is needed of the current state of knowledge on education and learning, encompassing their entire complexity: goals of current education systems and their alignment with today’s societal needs, the sociopolitical as well as education-specific contexts in which education is embedded, and the state-of-the-art insights into students’ learning experiences drawn from both the education and learning sciences including new insights from neuroscience. The challenge is to bring together different streams of knowledge that have been generated by different communities working on common areas, but yet have not drawn and built on each other’s work.

Addressing these key questions and challenges is exactly the aim of the International Scientific and Evidence-based Education Assessment (ISEE Assessment), where we take a multi-perspective, multicultural and multidisciplinary approach to advance rethinking the education agenda. The ISEE Assessment will contribute directly to the United Nations Educational Scientific and Cultural Organization (UNESCO) global “Futures of Education” initiative which was launched at the United Nations General Assembly in 2019.

The term “Assessment” here refers to a critical evaluation of the state of existing knowledge on education and learning by a team of independent experts drawn from a broad range of relevant disciplines and from across the world, interacting with key stakeholders in education. This knowledge will primarily be drawn from peer-reviewed scientific literature, but will also include credible grey literature. Importantly, the assessment will achieve a synthesis across disciplines by ongoing deliberative discussions across the team and stakeholders throughout the project, and by addressing overarching key questions and translating these answers into policy-relevant recommendations. In addition, this exercise will highlight gaps in knowledge and identify potential future research agendas.

To be clear, the ISEE Assessment is of a very different nature than international large-scale student assessments such as the Programme for International Student Assessment (PISA). Assessments like the one we propose here have proved extremely fruitful in other domains^4^ to synthesize information available from a wide range of disciplines. This has never before been performed for education.

## Education: an integrated approach

**Fig. 1:**
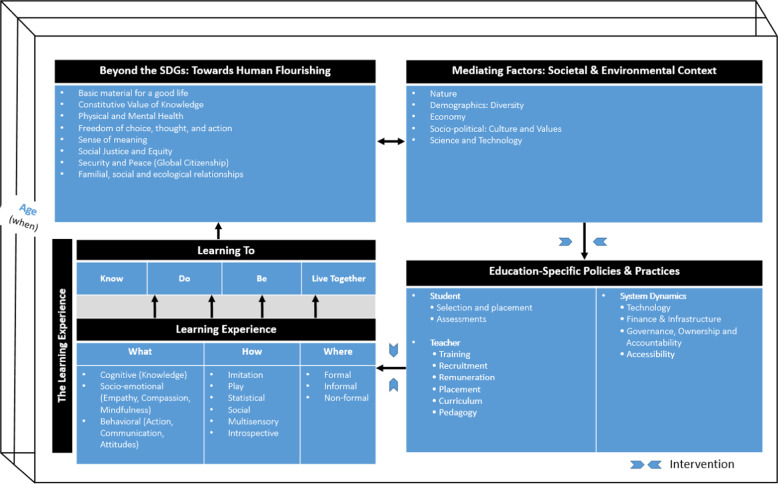
The ISEE Assessment Conceptual Framework (CF) of life-long learning. The CF shows how societal and environmental contexts (Box 2, top right) has an influence on educational policies and practices (Box 3, bottom right) which in turn influence the “What”, “Where”, “How” and “When” we learn (Box 4, bottom left). The four pillars of learning: Learning to Know; Learning to Do; Learning to Be; and Learning to Live Together (*19;* Box 4) are used as benchmarks for evaluating learning success which contribute to human flourishing (Box 1, top left) thereby providing an educational and learning lens at the individual level as a unifying thread to achieve the 17 Sustainable Development Goals (SDGs)^20^ that aim for a peaceful and sustainable world.

Developing a conceptual framework is an essential first step in approaching an assessment of this nature^4^. The conceptual framework presented in Fig. 1 captures the key inter-linkages within the education system, which will be assessed, and will guide the assessment.

First, we assess what we learn and its implications for education and learning (Box 4 in Fig. 1). We will evaluate

i. If there is purely a focus on knowledge acquisition—the cognitive or intellectual dimension of learning or broader to include the social and emotional dimensions. Emerging insights from the learning sciences, including neuroscience, emphasize the inherently inter-connectedness across the cognitive and the social, emotional and embodied dimensions of development and learning^5–8^;
ii. How contextual factors (e.g., cultural, political, environmental, technological) have influenced what we learn or need to learn (Box 2 in Fig. 1); and
iii. If what we learn in our current education systems will be sufficient to meet the challenges of the 21st century.

Second, we assess how we learn and its impact on education and learning. Education studies have long established the importance of forms of pedagogy for learning^9^, recognizing the centrality of the pedagogic device^10^ and children’s learning to learn^11^. Recently, pedagogy has evolved with many new methods such as gaming and learning through social interaction and play, as well as through contemplative practices that enhance sensual, emotional, and mental awareness^12^. Furthermore, over the last two decades, a rich body of information has been produced about how learning happens at the brain and behavioral levels, including individual differences and environmental influences on learning^13,14^. The educational implications of claims derived from research from these various disciplines have yet to be evaluated in an integrated manner relative to the learning experiences and practices that exist.

Third, we assess where we learn and its impact on education and learning. We evaluate the interplay between the formal (e.g., at school), non-formal (e.g., at work) and informal (i.e., unintentional such as via peer interactions) learning settings, face-to-face and online learning and the impact it has on learning. An urgent example is the recent shift to online learning forced by the Covid-19 pandemic and its implications. But also the increasingly significant phenomenon of ’shadow education’ (or private supplementary tutoring) in many societies, its causes and its implications for formal schooling, the learning experience and social inequality are important to assess^15^.

Fourth, we will assess when we learn and its impact on education and learning. Debate over the correct timing for formal education is longstanding, as is concern over the capacity of formal age-based schooling to accommodate diversity amongst learners. Studies from developmental psychology and neuroscience have revealed how the ease of learning varies with age, from infancy to old age^16,17^. Being offered the right inputs at the right time may improve learning, but at the same time, schooling conditions and (cultural) contexts may cause great variation in what is being taught when (Boxes 2 and 3 in Fig. 1). What implications might such insights have for the timing of interventions and design of school curricula? These are key questions many policy makers need answers to when redesigning their educational and learning policies.

### What will the ISEE Assessment provide?

First, it will provide an understanding of how our social, economic and political systems influence and are influenced by our education systems (the inter-dependent link between Box 2 and Box 3 in Fig. 1). We will examine how these contextual factors are related to diverse conceptions of the purpose of education (the inter-dependent link between Box 1 and Box 2). For example, the assessment will report on how economic policies, labor market pressures, and politics have influenced curriculum development, approaches to student assessment^18^, and competition for credentials across various global contexts.

Second, we will use the four pillars of education: (i) learning to Know, (ii) learning to Do, (iii) learning to Be, and learning to Live Together^19^ as benchmarks to analyze how contextual factors have influenced and been influenced by the educational aims and practices that these pillars encompass (the inter-linkages between Box 3 and Box 4 in Fig. 1). At the same time, we will assess the relationship between the “What”, “How”, “Where” and “When” of learning and the pillars of education in the light of state-of-the-art evidence from the science of learning, and studies of the socio-economic, environmental and other challenges we face today.

Third, we will assess how the pillars have contributed toward the conception of human flourishing and the interdependencies across the “What”, “Where”, “How” and “When” toward these pillars of education (inter-linkages between Box 4 and Box 1).

## Conclusion

There is an increasing recognition by policymakers that education and learning policies should be guided by science and evidence. The recent New Educational Policy released by the Government of India is a case in point. By synthesizing the state of existing knowledge on education and learning across disciplines and regions of the world, the ISEE Assessment will generate the information and recommendations needed taking into account what works, what does not and where more research is needed to guide and support policy-making beyond the 2030 education agenda. Moreover, the assessment comes at an opportune time when the world is reeling from the devastating impacts from the coronavirus that has as of March 2020 put about 1.53 billion children out of school. The sudden shift to online and digital technology poses many questions to educators and policy makers. Many questions that this assessment addresses will be useful guides in future crises and challenges.
